# Characterizing Emotion Recognition and Theory of Mind Performance Profiles in Unaffected Siblings of Autistic Children

**DOI:** 10.3389/fpsyg.2021.736324

**Published:** 2022-02-24

**Authors:** Mirko Uljarević, Nicholas T. Bott, Robin A. Libove, Jennifer M. Phillips, Karen J. Parker, Antonio Y. Hardan

**Affiliations:** ^1^Faculty of Medicine, Dentistry, and Health Sciences, Melbourne School of Psychological Sciences, University of Melbourne, Melbourne, VIC, Australia; ^2^Department of Psychiatry and Behavioral Sciences, School of Medicine, Stanford University, Stanford, CA, United States; ^3^Department of Psychology and Counseling, School of Psychology and Public Health, La Trobe University, Melbourne, VIC, Australia; ^4^Department of Medicine, Clinical Excellence Research Center, Stanford University School of Medicine, Stanford, CA, United States; ^5^PGSP-Stanford Consortium, Department of Psychology, Palo Alto University, Palo Alto, CA, United States

**Keywords:** theory of mind, autism spectrum disorder, unaffected siblings, broader autism phenotype, emotion recognition

## Abstract

Emotion recognition skills and the ability to understand the mental states of others are crucial for normal social functioning. Conversely, delays and impairments in these processes can have a profound impact on capability to engage in, maintain, and effectively regulate social interactions. Therefore, this study aimed to compare the performance of 42 autistic children (Mage = 8.25 years, *SD* = 2.22), 45 unaffected siblings (Mage = 8.65 years, *SD* = 2.40), and 41 typically developing (TD) controls (Mage = 8.56 years, *SD* = 2.35) on the Affect Recognition (AR) and Theory of Mind (TOM) subtests of the Developmental Neuropsychological Assessment Battery. There were no significant differences between siblings and TD controls. Autistic children showed significantly poorer performance on AR when compared to TD controls and on TOM when compared to both TD controls and unaffected siblings. An additional comparison of ASD, unaffected sibling and TD control subsamples, matched on full-scale IQ, revealed no group differences for either AR or TOM. AR and TOM processes have received less research attention in siblings of autistic children and remain less well characterized. Therefore, despite limitations, findings reported here contribute to our growing understanding of AR and TOM abilities in siblings of autistic children and highlight important future research directions.

## Introduction

Impairments in social functioning are a hallmark diagnostic feature of Autism Spectrum Disorder (American Psychiatric Association, 2013). These impairments have a pervasive negative impact across all aspects of functioning and lead to negative long-term outcomes (Mundy et al., 2009; Leekam, 2016). Both original clinical observations by Kanner (1943) and subsequent empirical evidence (Piven et al., 1997; Bailey et al., 1998; Bishop et al., 2006; Sucksmith et al., 2011; Ruzich et al., 2016) have demonstrated that, in addition to high rates of recurrence of the categorical ASD diagnosis, family members of autistic individuals exhibit subclinical levels of ASD related traits. This presence of traits characteristic of ASD, but exhibited to a lesser degree, has been termed the Broader Autism Phenotype (BAP; Piven et al., 1997; Bolton et al., 1998). Common neurobiological mechanisms have been suggested to underpin intermediate and fully manifested clinical phenotypes (Beauchaine and Constantino, 2017; Constantino, 2018), therefore, careful characterization of social functioning among unaffected relatives has the promise to lead to a better understanding of social impairments in ASD.

The capacity to perceive and interpret emotional states of others, communicated through the face, body, and tone of voice, referred to as affect recognition (AR; Herba and Phillips, 2004), and to make judgments and/or attributions about the mental state of oneself and others, commonly referred to as Theory of Mind (TOM; Happe et al., 2017), are among key components underpinning the ability to successfully navigate the complexities of the social world. A substantial body of work has demonstrated impairments in both AR and TOM in individuals diagnosed with ASD. A meta-analysis of 48 studies of AR (Uljarević and Hamilton, 2013) has established difficulties in this domain in ASD with medium effect size after the correction for publication bias. Similarly, meta-analysis by Chung et al. (2014) reported large effect sizes for both impairments in linguistic-contextual TOM tasks and the Reading the Mind in the Eyes task. Surprisingly, considering the substantial body of literature exploring other aspects of BAP, AR and TOM remain less well characterized in family members of autistic individuals, particularly in siblings. Studies to date have indicated impairments in both AR and TOM (Baron-Cohen and Hammer, 1997; Palermo et al., 2006; Gokcen et al., 2009; Kadak et al., 2014; but see Sucksmith et al., 2013) in parents of autistic individuals. With regards to AR and TOM abilities in siblings, findings have thus been inconsistent, with studies suggesting both impairments (Dorris et al., 2004; Oerlemans et al., 2013, 2014) and intact performance (Ozonoff et al., 1993; Bölte and Poustka, 2003; Shaked et al., 2006; Holt et al., 2014).

Emotion recognition skills and the ability to understand the mental states of others are crucial for normal social functioning. Conversely, delays and impairments in these processes can have a profound impact on social development, limiting the individual’s learning about other people’s emotions and mental states, thus impairing their capability to engage in, maintain, and effectively regulate social interactions. These two processes have also been highlighted by the National Institute of Mental Health’s Research Domain Initiative (RDoC) as two constructs with potentially distinct neurobiological substrates and as useful candidates for understanding variation in the social abilities, irrespective of the primary diagnostic status (Insel et al., 2010; National Institute of Mental Health, 2012). Therefore, the current investigation focused on characterizing the AR and TOM profiles among autistic children and their unaffected siblings in comparison to TD controls. We utilized the AR and the TOM tasks of the Developmental Neuropsychological Assessment Battery’s (NEPSY-II; Korkman et al., 2007) Social Perception scale. These two tasks enable detailed insight into the children’s ability to recognize and discriminate basic emotions of happiness, sadness, anger, fear and disgust, expressed through facial stimuli, and the ability to understand both the other person’s point of view and the relationship between specific emotions and social situations across diverse social contexts.

## Methods

### Participants

Forty-two autistic children (39 males; *M_*age*_* = 8.25 years, *SD* = 2.22), 45 unaffected siblings (26 males; *M_*age*_* = 8.65 years, *SD* = 2.40), and 41 typically developing (TD) controls (28 males; *M_*age*_* = 8.56 years, *SD* = 2.35) aged 5–12 years participated in this study. Participants were recruited as part of a larger study that focused on the relationships between oxytocin and social functioning, among autistic children, their siblings, and TD controls (Parker et al., 2014) but did not explore the differences in behavioral performance across groups. Autistic children and their unaffected siblings were primarily recruited through the Autism and Developmental Disorders Research Registry, and the Autism and Developmental Disorders Clinic, at Stanford University. Autistic participants met the following inclusion criteria: (1) diagnosis of ASD through DSM-IV diagnostic criteria and expert clinical evaluation, and confirmed through the Autism Diagnostic Interview-Revised (ADI-R; Rutter et al., 2003), and the Autism Diagnostic Observation Schedule (ADOS; Lord et al., 2012); and (2) absence of any neurological and genetic disorders (e.g., tuberous sclerosis or Fragile X syndrome). Siblings were included if they had no evidence of ASD based on clinical evaluation and the scores on the Social Responsiveness Scale-Second Edition (SRS-2; Constantino and Gruber, 2012). TD children were recruited through advertisements posted online (e.g., Parent Listservs,^1^) or hardcopy in the surrounding community (e.g., pediatrician offices, shopping malls) and had no present or lifetime history of psychiatric disorders.

### Measures

*Cognitive Functioning:* the Stanford-Binet Intelligence Scales—5th edition (SB5; Roid, 2003) is a test of overall cognitive development which evaluates verbal and non-verbal reasoning. It provides non-verbal intelligence (NVIQ) and verbal intelligence (VIQ) sub-scale scores, which together provide a full-scale intelligence quotient (FSIQ) score.

Affect Recognition (AR): the AR subscale of the NEPSY-II (Korkman et al., 2007) Social Perception scale was used to explore children’s s ability to recognize and discriminate facial expressions of six basic emotions (happy, sad, anger, fear, disgust, and neutral) presented through four separate tasks. The first three tasks require the participant to select two out of three (task 1), one out of four (task 2) and two out of four (task 3) photographs that match the target emotion expression (presented in a photograph of children’s face). The fourth task presents a child with a photo of facial emotion expression for five seconds, after which the child is asked to point two out of six photographs that match the emotion previously presented. Scores across these four tasks are summed to provide a total AR score.

*Theory of Mind (TOM):* the TOM subscale of the NEPSY-II was used. It consists of (1) the verbal task which combines elements of first- and second-order false belief, double deception and figurative language comprehension, and (2) the contextual task designed to evaluate an individual’s understanding of the relationship between specific emotions and social situations that diverse social contexts elicit. Scores across tasks are added to produce a total TOM score.

This study was approved by the Stanford University Institutional Review Board, and all participants and their families provided informed consent prior to the initiation of study procedures. Assent was also obtained from children 7 years of age and older when appropriate.

### Data Analysis

All analyses were run using IBM SPSS Statistics 24.0 for Mac (IBM Corp., Armonk, NY). Group differences on each measure of social cognition were evaluated using ANOVA with 5,000 resamples bootstrapping to provide more robust statistics (Tabachnick and Fidell, 2014). All analyses were supplemented with relevant effect sizes (overall ANOVA models with *Partial* η^2^, *post hoc* comparisons with Cohen’s d, and χ^2^*-*tests with *Phi*). For *Partial* η^2^, value of 0.01 indicates small, of 0.06 medium, and of 0.14 and higher large effect size. For Cohen’s d, value of 0.2 indicates small, of 0.5 medium, and of 0.8 or higher large effect size. For Phi, value of 0.1 indicates small, of 0.3 medium and of 0.5 or higher large effect size.

## Results

Sample characteristics of ASD, unaffected siblings, and TD groups are summarized in Table 1. There were no significant group differences in age. As expected, autistic children had significantly higher SRS T total scores (*F* = 168.05, *p* < 0.001, *Partial* η^2^ = 0.73), significantly lower FSIQ scores (*F* = 36.19, *p* < 0.001, *Partial* η^2^ = 0.37) and significantly higher proportion of males (χ^2^ = 14.03, *p* = 0.001, *Phi* = 0.33) than both unaffected siblings and TD controls, who in turn did not differ on SRS T total scores, FSIQ, or sex distribution.

**TABLE 1:** Participant characteristics.

Characteristics	ASD (a)	Siblings (b)	TD Controls (c)	Statistics
**N**	42	45	41	NA
**Age** M (*SD*) years	8.25 (2.22)	8.65 (2.40)	8.56 (2.35)	*F* = 0.35, *p* = 0.71, *Partial* η^2^ = 0.006
**Sex** M/F	39/3	26/19	28/13	χ^2^ = 14.03, *p* = 0.001, *Phi* = 0.33 *Post hoc*: a > b ≈ c
**SRS-2 T score** M(*SD*)	77.88 (12.18)	43.61 (8.33)	45.76 (7.78)	*F* = 168.05, *p* < 0.001, *Partial* η^2^ = 0.73 *Post hoc*: a > b ≈ c
**FSIQ** M(*SD*)	82.36 (27.62)	107.51 (12.68)	114.83 (9.49)	*F* = 36.19, *p* < 0.001, *Partial* η^2^ = 0.37 *Post hoc*: a < b ≈ c

*ASD, Autism Spectrum Disorder; FSIQ, Full Scale Inteligence Quotient; SRS-2, Social Responsiveness Scale 2nd edition; TD, typically developing.*

**FIGURE 1:**
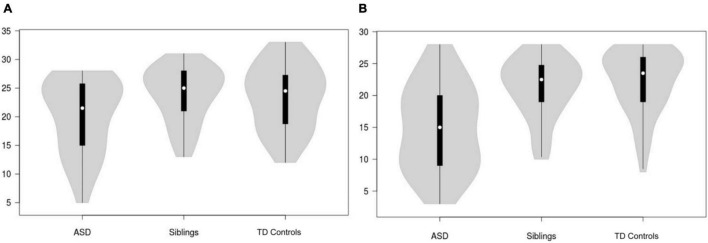
Violin plots showing distributions of NEPSY-II affect recognition **(A)** and theory of mind **(B)** task scores across ASD, unaffected siblings and TD controls. ASD, Autism Spectrum Disorder; TD, typically developing.

Distributions of AR and TOM NEPSY-II subscale scores across autistic children, unaffected siblings, and TD controls are shown in Figures 1A,B, respectively. There were significant group differences for both AR (*F* = 3.49, *p* = 0.033, *Partial* η^2^ = 0.06) and TOM (*F* = 19.10, *p* < 0.001, *Partial* η^2^ = 0.25) scores. *Post hoc* comparisons indicated that for AR, autistic children had significantly lower scores (poorer performance) when compared to TD controls [*p* = 0.042, 95% bootstrapped confidence intervals (95% bCI): -6.53 -0.09, Cohen’s *d* = 0.55]; the difference with unaffected siblings did not reach statistical significance. For TOM, autistic children had significantly lower scores than both TD controls (*p* < 0.001, bCI: -9.24 -3.37, Cohen’s *d* = 1.23) and unaffected siblings (*p* < 0.001, bCI: -10.28 -4.24, Cohen’s d = 1.08). Unaffected siblings and TD controls did not differ on AR or TOM scores. Given the significantly lower FSIQ in autistic children, a follow-up comparison was conducted in a subsample of autistic children, unaffected siblings, and TD controls matched on the FSIQ. Analysis of this comparison revealed no group differences for either AR (*F* = 0.36, *p* = 0.70, *Partial* η^2^ = 0.008) nor for TOM (*F* = 2.98, *p* = 0.057, *Partial* η^2^ = 0.06). Further, linear models indicated that after controlling for the FSIQ, the effects of the group were no longer significant for either AR (*F* = 5.56, *p* = 0.005, group: *t* = 0.88, *p* = 0.38, FSIQ: *t* = 2.29, *p* = 0.023) or TOM (*F* = 21.13, *p* < 0.001, group: *t* = 1.05, *p* = 0.29, FSIQ: *t* = 5.88, *p* < 0.001).

## Discussion

This study aimed to provide a detailed characterization of two key components of social cognition, AR and TOM, in a sample of autistic children, their unaffected siblings and TD controls. In the present study, we found that unaffected siblings did not differ from TD controls on AR and TOM tasks. Although in a full sample comparison, autistic children showed significantly poorer performance on the NEPSY-II AR and ToM subtests when compared to siblings and TD controls, the follow-up comparison showed that a subgroup of autistic children matched on the FSIQ with the other two groups did not differ on either of the tasks. This observation was confirmed with full linear models. Thus, it was FSIQ, rather than group membership, that had more important effects on the performance on both AR and TOM tasks. This finding is in line with the previous studies that also did not report significant group differences between ASD and FSIQ matched control groups (e.g., Castelli, 2005; Da Fonseca et al., 2009; Jones et al., 2011).

Our findings are in line with Bölte and Poustka (2003) who found no difference in AR between a sample of siblings of autistic children when compared to controls and with studies showing intact performance on the Reading Mind in the Eyes (Holt et al., 2014) and contextual TOM tasks (Ozonoff et al., 1993; Shaked et al., 2006) in unaffected siblings. However, findings reported here are not consistent with several other studies suggesting impairments on tasks tapping into these social processes (TOM: Dorris et al., 2004; AR: Oerlemans et al., 2014 [of note: AR impairments were limited only to recognition of happiness from visual and fear from auditory domain; AR performance was intact for other emotions]). Although our study is the first to utilize NEPSY-II AR and TOM tasks in unaffected siblings of autistic children, the AR task used here is similar to tasks used by Bölte and Poustka (2003) and Oerlemans et al. (2014), and the TOM scale encompasses similar elements included in prior TOM paradigms. Furthermore, studies by Dorris et al. (2004) and Holt et al. (2014) that used identical TOM tasks (Reading Mind in the Eyes), in similarly aged samples of siblings of autistic children, have reported opposite findings. Therefore inconsistencies in the findings to date are unlikely to be attributable to the differences in tasks used across the studies. Importantly, Dalton et al. (2007) reported that although a sample of unaffected siblings of autistic children (*N* = 10) did not differ from TD controls in terms of behavioral performance on facial recognition tasks, they nevertheless showed a pattern of gaze fixations and atypical activation of the fusiform gyrus that was similar to their siblings and distinct from TD controls. Although limited by very small sample size, study by Dalton and colleagues highlights the importance of incorporating more implicit performance measures such as eye-tracking as well as indices of underlying neurobiological underpinnings in order to gain full insight into the AR and TOM across different units of analyses. The importance of investigating shared and distinct neurobiological mechanisms is further emphasized by a relatively recent study by Parker et al. (2014) who reported that plasma oxytocin concentration was positively associated with the TOM performance across ASD, unaffected sibling, and TD control groups.

Previous research has demonstrated that elevated levels of ASD traits are more common among individuals from multiplex than from simplex ASD families (Constantino et al., 2006; Gerdts et al., 2013). This has led to the notion of distinct genetic transfer mechanisms depending on the multiplex vs. simplex status (Bernier et al., 2012). Our study has specifically focused on simplex families which can potentially account for the intact performance on AR and TOM tasks in the siblings. In addition, heterogeneity is a hallmark feature of ASD, as demonstrated by our findings that a subset of autistic children matched on age and FSIQ with both TD controls and unaffected siblings showed comparable performance on both NEPSY-II tasks. This finding is consistent with a number of studies that did not find evidence for impaired AR (Ozonoff et al., 1990; Piggot et al., 2004; Castelli, 2005; Spezio et al., 2007; Loveland et al., 2008; Da Fonseca et al., 2009; Lacroix et al., 2009; Jones et al., 2011) or TOM (Ponnet et al., 2004; Fisher et al., 2005; Scheeren et al., 2010) among autistic individuals. Given this, it is not surprising that pronounced individual differences have also been reported among family members of individuals with ASD. For example, two studies by Losh and Piven (2007) and Losh et al. (2009) suggest that impairments in TOM skills might be only constrained to a subgroup of parents that exhibit elevated levels of ASD traits and a recent study by Ruzich et al. (2016) has reported the existence of low and high severity clusters [based on the Autism Spectrum Quotient (Baron-Cohen et al., 2001) scores] in a large sample of siblings. Therefore, it will be important for future research to further characterize potential distinct subgroups among siblings of autistic children based on the unique profiles of strengths and weaknesses across a comprehensive battery or AR and TOM tasks.

The findings reported here should be considered in light of several limitations. While this study included AR and TOM measures, only one measure per construct was administered, therefore, a more comprehensive assessment is needed to provide increased sensitivity. Further, it is possible that NEPSY AR and TOM tasks are not sufficiently sensitive to capture subtle differences in AR and TOM abilities. These more subtle differences might be better captured by more ecologically valid tasks and could certainly have significant negative impact on real world social functioning abilities. Therefore, it will be important for future studies to include a broader range of tasks that include more complex emotions and more subtle/low-intensity stimuli, non-facial stimuli including body expressions and prosody, as well as explore recognition abilities across both lab-based and more ecologically valid contexts and settings. Although we have utilized robust statistics and considered effect sizes for each of the comparisons, the sample size was nevertheless modest. This, in addition to the focus on siblings from simplex families, highlights the need for further work in larger and more heterogeneous samples. Finally, although sex distribution in specific subgroups was in line with the sex distribution previously reported in ASD and TD samples, low number of female participants in the ASD group did not allow this study to explore potential effects of sex. Thus, it will be crucial for future studies to specifically address this question, and if sufficiently powered, also explore potential moderating effects of age and FSIQ on the potential sex differences in AR and TOM abilities.

The ability to recognize and interpret emotions and to attribute mental states to oneself and others to predict and explain behaviors are two key basic components of social functioning and areas of particular weakness among the majority of autistic individuals. However, these two social processes have received less research attention in siblings of autistic individuals and remain less well characterized. Therefore, despite noted limitations, findings reported here contribute to our growing understanding of AR and TOM abilities in siblings of autistic children, and highlight important avenues for future research.

## Data Availability Statement

The raw data supporting the conclusions of this article will be made available by the authors, upon request without undue reservation.

## Ethics Statement

This study was approved by the Stanford University Institutional Review Board, and all participants and their families provided informed consent prior to the initiation of study procedures. Assent was also obtained from children 7 years of age and older when appropriate. Written informed consent to participate in this study was provided by the participants’ legal guardian/next of kin.

## Author Contributions

AH and KP designed the study. AH, KP, JP, and NB collected the data. MU and NB had full access to the data and conducted the analyses. MU, NB, KP, and AH drafted the initial manuscript. All authors critically reviewed, provided feedback on the initial version of the manuscript, and approved the final version of the manuscript.

## Conflict of Interest

The authors declare that the research was conducted in the absence of any commercial or financial relationships that could be construed as a potential conflict of interest.

## Publisher’s Note

All claims expressed in this article are solely those of the authors and do not necessarily represent those of their affiliated organizations, or those of the publisher, the editors and the reviewers. Any product that may be evaluated in this article, or claim that may be made by its manufacturer, is not guaranteed or endorsed by the publisher.

## Footnotes

1
www.craigslist.org
